# Comparison of outcomes after topography-modified refraction versus wavefront-optimized versus manifest topography-guided LASIK

**DOI:** 10.1186/s12886-020-01459-0

**Published:** 2020-05-14

**Authors:** Jaeryung Kim, Sung-Ho Choi, Dong Hui Lim, Gil-Joong Yoon, Tae-Young Chung

**Affiliations:** 1 Department of Ophthalmology, Samsung Medical Center, Sungkyunkwan University School of Medicine, Seoul, 06351 Republic of Korea; 2 BALGEUN-EYE21 Operation Center, Gwangju, 61932 Republic of Korea

**Keywords:** LASIK, Topography-modified refraction LASIK, Topography-guided LASIK, Wavefront-optimized LASIK, Astigmatic overcorrection

## Abstract

**Background:**

To compare the outcomes of myopia and myopic astigmatism corrected with topography-modified refraction laser in situ keratomileusis (TMR-LASIK), wavefront-optimized (WFO) LASIK, and topography-guided (TG) LASIK with a correction target based on the manifest refraction (manifest TG-LASIK).

**Methods:**

This observational, retrospective cohort study included patients who underwent LASIK using the WaveLight® EX500 excimer laser to correct myopia and myopic astigmatism between August 2016 and July 2017. Patients who underwent TMR-LASIK (85 patients), WFO-LASIK (70 patients), or manifest TG-LASIK (40 patients) were enrolled, and only one eye from each patient was analyzed. All participants underwent measurement of the uncorrected distance visual acuity (UDVA), best-corrected distance visual acuity (BCVA), manifest refraction, vector analysis of astigmatic change, corneal topography, and corneal wavefront analysis at baseline and at every posttreatment visit.

**Results:**

Three months postoperatively, a UDVA of 0.0 logMAR or better and manifest refraction spherical equivalent (MRSE) within ±0.5 diopters (D) did not differ across the TMR-, WFO-, and manifest TG-LASIK groups. However, the residual cylinder in the TMR group was significantly larger than that in the WFO and manifest TG groups. The magnitude of error in the TMR group measured using astigmatism vector analysis was significantly higher than that in the WFO and manifest TG groups.

**Conclusions:**

Although these three LASIK platforms achieved the predicted surgical outcomes, TMR-LASIK overcorrected astigmatism and showed a higher residual postoperative astigmatism compared with WFO- and manifest TG-LASIK.

## Background

Customized refractive surgery based on corneal topography has been widely used to correct myopia, astigmatism, and higher-order aberrations (HOAs). It has also shown satisfactory surgical outcomes and common post-laser in situ keratomileusis (LASIK) symptoms including light sensitivity, glare, and halos [1, 2]. To determine the most effective and safest treatment algorithm for refractive surgery, previous landmark studies have shown that topography-guided (TG) ablation resulted in better refractive and visual outcomes and fewer HOAs compared with wavefront-optimized (WFO) ablation [3–5].

LASIK surgery using topography-modified refraction (TMR) was introduced by Kanellopoulos based on the differences in the amount and axis of refractive astigmatism (RA) from those of corneal astigmatism (CA) [6]. In his study, a topographic adjustment was applied relative to the amount and axis of astigmatism, and TMR-LASIK showed better visual and refractive outcomes compared with TG-LASIK with a correction target based on the manifest refraction (manifest TG-LASIK) [6]. In our experience with TMR-LASIK, we have observed a generalized tendency toward overcorrection. Therefore, the primary purpose of this study was to evaluate whether TMR-LASIK overcorrected astigmatism and to compare surgical outcomes following surgery with TMR-, WFO-, and manifest TG-LASIK.

## Methods

This observational retrospective cohort study included patients undergoing LASIK to correct myopia and myopic astigmatism performed by two experienced surgeons (S.H.C. and G-.J.Y.) between August 2016 and July 2017. Patients who underwent TMR-LASIK (85 patients), WFO-LASIK (70 patients), or manifest TG-LASIK (40 patients) were enrolled, and only the right eye of each patient was analyzed in this study to avoid possible inter-eye correlations. This study was approved by the institutional review board (IRB) of the Samsung Medical Center (IRB no. 2019–01-090), and all work adhered to the tenets of the Declaration of Helsinki.

We included virgin eyes of patients with preoperative refractive error between − 0.50 and − 7.50 diopters (D) of spherical myopia and between 0.00 and 3.00 D of astigmatism and distance visual acuity correctable to 0.1 logMAR or better. The exclusion criteria for the LASIK operation were as follows: eyes with a significant dry eye, cataracts, or corneal scarring; a history of recurrent corneal erosion and keratoconus; estimated postoperative residual stromal bed thickness less than 250 μm; retinal and optic nerve disease; and systemic conditions including autoimmune disease, pregnancy, and lactation. The surgical protocol used for each patient was selected according to the surgeon’s subjective determination on a case-by-case basis.

All eyes were evaluated preoperatively for best-corrected distance visual acuity (BCVA) using spectacles. Preoperative examinations included manifest refraction, cycloplegic refraction with Mydrin-P (Santen, Osaka, Japan; tropicamide 0.5% and phenylephrine HCl 0.5%), and corneal topography assessment utilizing the WaveLight® Topolyzer Vario™ (Alcon, Fort Worth, TX, USA). For contact lens users, discontinuation of lens wear was requested prior to screening for either 2 weeks (soft contact lenses) or 4 weeks (rigid gas-permeable contact lenses). Manifest refraction, corneal topography assessment, and wavefront analysis were performed at two separate visits to ensure refractive stability.

For manifest TG-LASIK, we followed the method described in the study conducted by the Food and Drug Administration (FDA) to approve the Contoura™ Vision software (Alcon): the correction target was based on the manifest refraction, with a target of emmetropia [2]. According to the previously-suggested TMR method [6], the treatment used in the eyes of the TMR group was modified to reflect the power and axis of the topographically measured cylinder measured by the WaveLight® Topolyzer Vario™. In both the TG and TMR groups, corneal topographies obtained by at least three consistent images were transferred to the Contoura™ Vision software to correct HOAs. The corneal flaps were created with a flap diameter of 9 mm and programmed thickness of 105 μm by using the WaveLight® FS200 femtosecond laser (Alcon). The ablation of the corneal stroma was conducted by the WaveLight® EX500 excimer laser (Alcon) in all eyes. Postoperative follow-up examinations were performed at 1 day, 1 week, 1 month, and 3 months. At all follow-up visits, measurements of UDVA, manifest refraction, corneal topography, and corneal wavefront analysis were performed by the same examiner who performed the preoperative assessments. As previously described [7, 8], the Alpins vector analysis was used to evaluate the change in astigmatism at 3 months postoperative using the Excel software (Microsoft, Redmond, WA, USA). Briefly, six key parameters were calculated: target-induced astigmatism (TIA), indicating the intended vector to change the cylinder; surgically-induced astigmatism (SIA), representing the actual astigmatic change achieved by surgery; difference vector (DV), meaning the difference between the achieved astigmatism and the target astigmatism; magnitude of error (ME), which is the arithmetic difference in SIA and TIA; correction index (CI), which is calculated by dividing SIA by TIA; and index of success (IOS), which is determined by dividing DV by TIA.

For continuous variables presented as the mean ± standard deviation (SD), the indicated *P*-values were obtained using one-way analysis of variance (ANOVA) followed by a post-hoc Bonferroni test or unpaired *t*-test. The categorical variables were described as proportions and were compared using the chi-square test or Fisher’s exact test. A *P*-value less than 0.05 was considered to indicate statistical significance. All calculations were performed using PASW Statistics 18 (SPSS, Inc., Chicago, IL, USA).

## Results

The study comprised 70 eyes in the WFO group, 40 eyes in the manifest TG group, and 85 eyes in the TMR group. Table 1 compares the preoperative parameters among the TMR, WFO, and manifest TG groups, showing that no preoperative parameters differed significantly among the three groups.

**Table 1:** Preoperative parameters

Parameter (mean ± SD)	WFO	Manifest TG	TMR	*P*-value
BCVA (logMAR)	−0.06 ± 0.07	−0.07 ± 0.07	−0.08 ± 0.06	.122
Sphere (D)	−4.01 ± 1.65	−4.31 ± 1.95	−3.52 ± 2.23	.085
Cylinder (RA; D)	−0.89 ± 0.55	−0.99 ± 0.70	−1.09 ± 0.69	.172
MRSE (D)	−4.45 ± 1.75	−4.81 ± 1.95	−4.06 ± 2.26	.142
Pachymetry (μm)	542.80 ± 27.24	534.95 ± 31.01	537.62 ± 25.49	.300
Flat keratometry (D)	42.66 ± 1.38	42.68 ± 1.29	42.64 ± 1.22	.986
Steep keratometry (D)	43.88 ± 1.41	43.85 ± 1.32	44.01 ± 1.32	.774
CA (D)	1.22 ± 0.63	1.17 ± 0.59	1.37 ± 0.63	.171
Magnitude difference between CA and RA (D)	0.33 ± 0.39	0.18 ± 0.36	0.33 ± 0.39	.146
Axis difference between CA and RA (°)	15.91 ± 20.46	18.60 ± 21.19	12.78 ± 18.52	.288

WFO = wavefront-optimized; TG = topography-guided; TMR = topography-modified refraction; D = diopters; BCVA = best-corrected distance visual acuity; logMAR = logarithm of the minimal angle of resolution; RA = refractive astigmatism; MRSE = manifest refraction spherical equivalent; CA = corneal astigmatism

### Refractive and visual outcomes

**Fig. 1:**
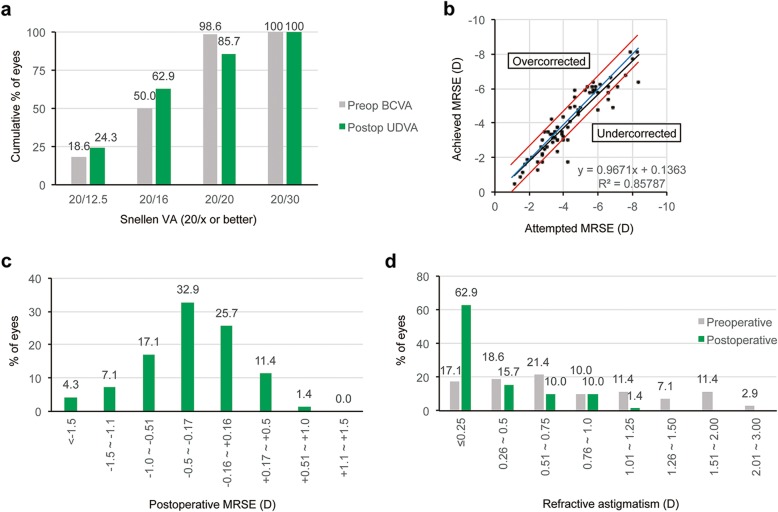
Refractive and visual outcomes in the wavefront-optimized group three months postoperative. (**a**) Cumulative distribution of uncorrected distance Snellen visual acuity (UDVA) compared with preoperative best-corrected distance visual acuity (BCVA). Forty-four (67.7%) and 60 (92.3%) eyes had a UDVA better than 20/16 and 20/20, respectively, at three months after surgery. (**b**) Scatter plots of attempted versus achieved manifest refraction spherical equivalent (MRSE). (**c**) Accuracy of MRSE. Forty-nine eyes (75.4%) had MRSE within ±0.75 D of emmetropia at three months after surgery. (**d**) Cumulative distribution of refractive astigmatism (RA) compared with the preoperative value. Thirty-seven (56.9%) and 48 (73.8%) eyes had an RA less than 0.25 D and 0.50 D, respectively, at three months after surgery

**Fig. 2:**
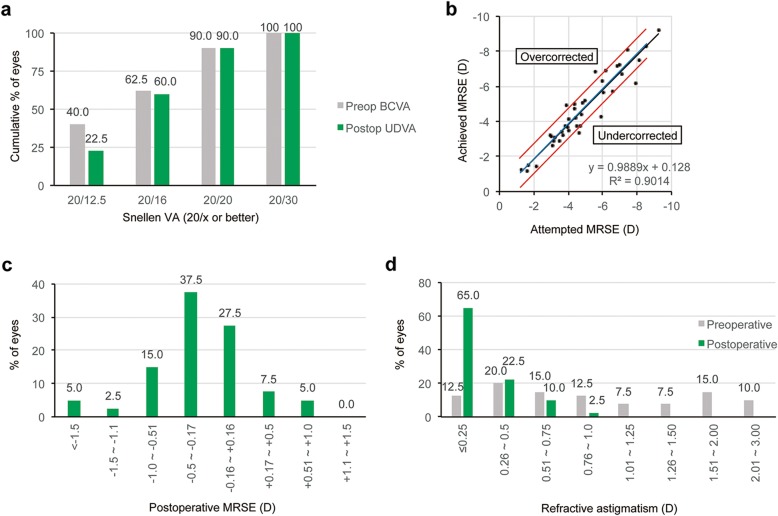
Refractive and visual outcomes in the manifest topography-guided group three months postoperative. (**a**) Cumulative distribution of uncorrected distance Snellen visual acuity (UDVA) compared with preoperative best-corrected distance visual acuity (BCVA). Twenty-four (60.0%) and 36 (90.0%) eyes had a UDVA better than 20/16 and 20/20, respectively, at three months after surgery. (**b**) Scatter plots of attempted versus achieved manifest refraction spherical equivalent (MRSE). (**c**) Accuracy of MRSE. Thirty-three eyes (82.5%) had MRSE within ±0.75 D of emmetropia at three months after surgery. (**d**) Cumulative distribution of refractive astigmatism (RA) compared with the preoperative value. Twenty-six (65.0%) and 35 (87.5%) eyes had an RA less than 0.25 D and 0.50 D, respectively, at three months after surgery

**Fig. 3:**
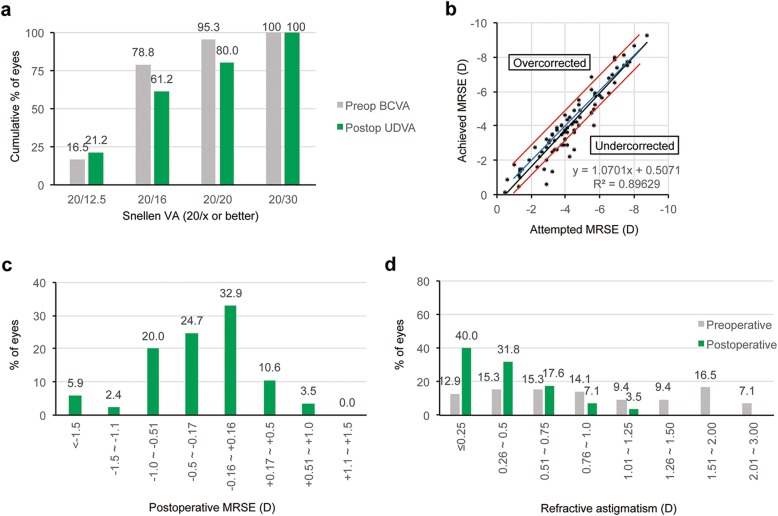
Refractive and visual outcomes in the topography-modified refraction group three months postoperative. (**a**) Cumulative distribution of uncorrected distance Snellen visual acuity (UDVA) compared with preoperative best-corrected distance visual acuity (BCVA). Fifty-two (61.2%) and 68 (80.0%) eyes had a UDVA better than 20/16 and 20/20, respectively, at three months after surgery. (**b**) Scatter plots of attempted versus achieved manifest refraction spherical equivalent (MRSE). (**c**) Accuracy of MRSE. Seventy-one eyes (83.5%) had MRSE within ±0.75 D of emmetropia at three months after surgery. (**d**) Cumulative distribution of refractive astigmatism (RA) compared with the preoperative value. Thirty-four (40.0%) and 61 (71.8%) eyes had an RA less than 0.25 D and 0.50 D, respectively, at three months after surgery

Figures 1, 2, and 3 show the refractive and visual outcomes of the WFO, manifest TG, and TMR groups, respectively. A UDVA of 20/20 or better was measured at 3 months after surgery in 85.7% of eyes in the WFO group, 90.0% of eyes in the manifest TG group, and 80.0% of eyes in the TMR group (*P* = .781; Figs. 1, 2, and 3a). The distribution of postoperative UDVA was not significantly different among the three groups (*P* = .881). The mean manifest refraction spherical equivalent (MRSE) at 3 months was − 0.29 ± 0.68 D in the WFO group, − 0.18 ± 0.64 D in the manifest TG group, and − 0.21 ± 0.69 in the TMR group (*P* = .630). The percentages of eyes having MRSE within ±0.5 D and ± 1.0 D of emmetropia measured at 3 months were 70.0 and 88.5% of eyes in the WFO group, 72.5 and 92.5% of eyes in the manifest TG group, and 68.2 and 91.7% of eyes in the TMR group (*P* = .888 and .724, respectively; Figs. 1, 2, and 3b). The distribution of postoperative MRSE at 3 months was not significantly different among the three groups (*P* = .777; Figs. 1, 2, and 3c and b). The mean postoperative RA at 3 months was − 0.35 ± 0.34 D in the WFO group, − 0.30 ± 0.27 D in the manifest TG group, and − 0.45 ± 0.35 D in the TMR group (*P* = .035). The distribution of postoperative RA at 3 months was significantly different among the three groups (*P* = .010; Figs. 1, 2, and 3d): the value in the TMR group was higher than that in both the WFO (*P* = .027) and manifest TG (*P* = .006) groups.

### Vector analysis of astigmatic change

We analyzed the change in the RA based on the Alpins method of vector analysis (Table 2). TIA and SIA in the TMR group were greater than those of the WFO group. However, there was no significant difference in TIA or SIA between the TMR and manifest TG groups. IOS was less than 1 in all groups, meaning that astigmatism decreased. Intriguingly, the ME in the TMR group (0.23 ± 0.39) was greater than that of the manifest TG (0.03 ± 0.31, *P* = 0.015) and the WFO (0.08 ± 0.34, *P* = 0.041) groups, indicating that there was a significantly larger amount of astigmatic overcorrection in the TMR group.

**Table 2:** Change in astigmatism based on the Alpins method of vector analysis

Parameter (mean ± SD)	WFO	Manifest TG	TMR	*P*-value	Post hoc (*P*-value)
TIA	0.90 ± 0.50	1.10 ± 0.74	1.17 ± 0.64	.035*	TMR > WFO (.032)
SIA	0.99 ± 0.61	1.13 ± 0.77	1.40 ± 0.75	.002*	TMR > WFO (.002)
DV	0.33 ± 0.31	0.29 ± 0.27	0.47 ± 0.35	.005*	TMR > WFO (.026), Manifest TG (.014)
ME	0.08 ± 0.34	0.03 ± 0.31	0.23 ± 0.39	.007*	TMR > WFO (.041), Manifest TG (.015)
CI	1.10 ± 0.46	1.09 ± 0.47	1.27 ± 0.51	.057	
IOS	0.50 ± 0.56	0.43 ± 0.53	0.53 ± 0.51	.607	

WFO = wavefront-optimized; TG = topography-guided; TMR = topography-modified refraction; TIA = target-induced astigmatism; SIA = surgically-induced astigmatism; DV = difference vector; ME = magnitude of error; CI = correction index; IOS = index of success. *Statistically significant (*P* < .05)

### Higher-order aberrations

Table 3 shows preoperative, three-month postoperative, and magnitude of surgically-induced corneal HOAs. Preoperative HOAs did not differ significantly among the three groups. At 3 months postoperative, HOAs including total HOA, coma, and spherical aberration increased in all groups. Compared with the WFO group, the TMR group showed significantly lower total HOA (*P* = < .001), coma (*P* = < .001), trefoil (*P* = .034), and spherical aberration (*P* = .002). On the other hand, there was no significant difference in trefoil or spherical aberration between the manifest TG group and the WFO group. Regarding the magnitude of surgically-induced HOAs, TMR-LASIK induced significantly lower total HOA (*P* = < .001), coma (*P* = < .001), and spherical aberration (*P* = .005) than did WFO-LASIK. Manifest TG-LASIK also induced significantly lower total HOA (*P* = .006) and coma (*P* = .010) than did WFO-LASIK. There was no significant difference in magnitude of surgically-induced HOAs between TMR- and manifest TG-LASIK.

**Table 3:** Preoperative, three-month postoperative, and magnitude of surgically-induced corneal HOAs

Parameter (Mean RMS value ± SD, μm)	WFO	Manifest TG	TMR	*P-*value	Post hoc (*P*-value)
Preoperative HOAs					
Total HOAs	0.41 ± 0.16	0.46 ± 0.20	0.44 ± 0.14	.201	
Coma	0.19 ± 0.09	0.22 ± 0.13	0.20 ± 0.11	.372	
Trefoil	0.15 ± 0.09	0.15 ± 0.10	0.14 ± 0.08	.791	
Spherical aberration	0.15 ± 0.06	0.13 ± 0.06	0.14 ± 0.06	.158	
Three months postoperative HOAs					
Total HOAs	0.76 ± 0.38	0.60 ± 0.30	0.55 ± 0.19	.000*	TMR < WFO (< .001), Manifest TG < WFO (.022)
Coma	0.37 ± 0.25	0.26 ± 0.18	0.24 ± 0.14	.000*	TMR < WFO (< .001), Manifest TG < WFO (.031)
Trefoil	0.16 ± 0.09	0.14 ± 0.09	0.12 ± 0.10	.040*	TMR < WFO (.034)
Spherical aberration	0.36 ± 0.25	0.31 ± 0.22	0.23 ± 0.16	.002*	TMR < WFO (.002)
Magnitude of surgically-induced HOAs					
Total HOAs	0.37 ± 0.41	0.14 ± 0.41	0.12 ± 0.25	.000*	TMR < WFO (< .001), Manifest TG < WFO (.006)
Coma	0.18 ± 0.26	0.05 ± 0.23	0.04 ± 0.18	.001*	TMR < WFO (.001), Manifest TG < WFO (.010)
Trefoil	0.01 ± 0.10	− 0.01 ± 0.09	− 0.02 ± 0.12	.233	
Spherical aberration	0.21 ± 0.25	0.19 ± 0.25	0.09 ± 0.17	.004*	TMR < WFO (.005)

HOAs = higher-order aberrations; RMS = root mean square; WFO = wavefront-optimized; TG = manifest topography-guided; TMR = topography-modified refraction. *Statistically significant (*P* < .05)

### Subgroup analysis of the TMR group

In addition to the fact that the correction target in TMR-LASIK is based on the corneal topographic astigmatism value, the aforementioned vector analysis finding of astigmatic overcorrection in the TMR group led us to investigate whether a certain relationship exists between the overcorrection of astigmatism and the magnitude of difference between preoperative CA and RA. To gain insight into this question, we divided the TMR group into two subgroups: Subgroup 1 having CA < RA (*n* = 16) and Subgroup 2 having CA ≥ RA (*n* = 69). Although there was no significant difference in MRSE or visual acuity between the two subgroups, the ME in subgroup 2 (0.28 ± 0.38) was significantly greater than that of subgroup 1 (0.04 ± 0.38), indicating that astigmatic overcorrection in TMR-LASIK mostly stemmed from the eyes in subgroup 2 (Table 4). In addition, the following parameters in the TMR group were found to be significantly correlated with ME: preoperative magnitude difference between CA and RA (Pearson’s *r* = 0.393, *P* = < .001), preoperative CA (*r* = 0.237, *P* = .036), and postoperative cylinder (*r* = − 0.646, *P* = < .001). To further determine parameters which have a significant impact on ME, a multivariable linear regression analysis was performed. All parameters that had been revealed by an univariable linear regression analysis to be significantly associated with ME were incorporated into the multivariable-adjusted linear regression analysis, and the preoperative magnitude difference between CA and RA (coefficient = 0.262, *P* = 0.002) and postoperative cylinder (coefficient = − 0.677, *P* = < 0.001) were identified as significant factors (Table 5).

**Table 4:** Subgroup analysis of the TMR group

Parameter (Mean ± SD)	Subgroup 1 (CA < RA)	Subgroup 2 (CA > RA)	*P* value
Preoperative			
BCVA (logMAR)	−0.07 ± 0.07	−0.07 ± 0.06	.843
Sphere (D)	−2.55 ± 3.57	− 3.74 ± 1.75	.210
Cylinder (RA; D)	−1.48 ± 0.69	−1.00 ± 0.66	.009*
MRSE (D)	− 3.29 ± 3.69	− 4.24 ± 1.77	.330
Flat keratometry (D)	42.95 ± 1.29	42.57 ± 1.20	.260
Steep keratometry (D)	44.09 ± 1.45	43.99 ± 1.30	.780
CA (D)	1.14 ± 0.62	1.42 ± 0.63	.111
Magnitude difference between CA and RA (D)	− 0.34 ± 019	0.43 ± 0.27	.000*
Axis difference between CA and RA (°)	19.3 ± 11.1	25.4 ± 16.3	.230
Postoperative 3 months			
UDVA (logMAR)	− 0.03 ± 0.11	−0.05 ± 0.11	.415
UDVA (% of logMAR 0.0 or better)	68.8	82.6	.296
Sphere (D)	0.14 ± 0.79	−0.01 ± 0.66	.440
Cylinder (D)	−0.52 ± 0.23	−0.44 ± 0.37	.297
MRSE (D)	−0.12 ± 0.80	−0.23 ± 0.67	.571
MRSE (% of D within ±0.5)	68.8	68.1	.999
MRSE (% of D within ±1.0)	93.8	91.3	.999
Change in astigmatism based on the Alpins method of vector analysis			
TIA	1.48 ± 0.69	1.09 ± 0.60	.027*
SIA	1.52 ± 0.61	1.37 ± 0.78	.470
DV	0.52 ± 0.23	0.46 ± 0.37	.568
ME	0.04 ± 0.38	0.28 ± 0.38	.027*
CI	1.16 ± 0.52	1.30 ± 0.51	.315
IOS	0.47 ± 0.39	0.55 ± 0.54	.591

TMR = topography-modified refraction; CA = corneal astigmatism; RA = refractive astigmatism; BCVA = best-corrected distance visual acuity; logMAR = logarithm of the minimal angle of resolution; MRSE = manifest refraction spherical equivalent; UDVA = uncorrected distance visual acuity; TIA = target-induced astigmatism; SIA = surgically-induced astigmatism; DV = difference vector; ME = magnitude of error; CI = correction index; IOS = index of success. *Statistically significant (*P* < .05)

**Table 5:** Multivariable linear regression analysis of parameters with statistically significant association with ME on univariable analysis in the TMR group

Parameter	Value	Coefficient	95% CI	*p*-value
Preoperative CA (D)				
Univariable analysis	1.37 ± 0.63	0.149	0.012–0.285	0.036*
Multivariable analysis		−0.003	−0.106 – 0.099	0.950
Preoperative magnitude difference between CA and RA (D)				
Univariable analysis	0.33 ± 0.39	0.392	0.187–0.597	< 0.001*
Multivariable analysis		0.262	0.102–0.422	0.002*
Postoperative cylinder (D)				
Univariable analysis	−0.45 ± 0.35	− 0.728	− 0.920 – − 0.536	< 0.001*
Multivariable analysis		− 0.677	− 0.855 – − 0.498	< 0.001*

ME = magnitude of error; TMR = topography-modified refraction; CA = corneal astigmatism; RA = refractive astigmatism. *Statistically significant (*P* < .05)

## Discussion

In this study, we observed higher residual postoperative astigmatism and a greater overcorrection of astigmatism in eyes of the TMR-LASIK group compared with the WFO- and manifest TG-LASIK groups even though the distributions of postoperative UDVA and MRSE were not different among the three groups. Regarding HOAs after surgery, TMR- and manifest TG-LASIK showed significantly lower induction of corneal HOAs compared with WFO-LASIK.

In the present study, WFO-, manifest TG-, and TMR-LASIK produced comparable visual outcomes. Consistent with the results of a meta-analysis of 718 eyes undergoing LASIK with one of the three United States FDA-approved platforms [9], the distribution of postoperative UDVA did not differ significantly between WFO-LASIK, manifest TG-LASIK, and TMR-LASIK groups in this study. On the other hand, in a previous contralateral-eye comparison of WFO- and TMR-LASIK [6], Kanellopoulos reported that the number of eyes that achieved postoperative UDVA values of 20/16 and 20/20 was significantly higher in TMR-LASIK than in WFO-LASIK. In another previous contralateral-eye comparison of WFO- and manifest TG-LASIK [4], Jain et al. also reported that eyes treated with WFO-LASIK had relatively worse visual outcomes than those treated with TG-LASIK.

Our results showed that the predictability of the refractive correction and visual outcomes did not differ significantly among WFO-, manifest TG-, and TMR-LASIK, similar with those of previous contralateral-eye comparisons of WFO-LASIK with manifest TG- or TMR-LASIK [4–6]. However, in this study, the mean value of postoperative RA was higher in TMR-LASIK, and TMR-LASIK showed a significantly-skewed distribution of postoperative RA toward higher astigmatic values than WFO- and manifest TG-LASIK. On the other hand, a previous comparison between WFO- and TMR-LASIK reported conflicting results with respect to RA [6]. When we performed vector analysis of astigmatic change to evaluate the reason for the difference in postoperative RA between our result and that of the previous study [6], TMR-LASIK significantly overcorrected astigmatism more than WFO- or manifest TG-LASIK, and the astigmatism values in the eyes of the TMR group with larger CA than RA were significantly more overcorrected compared with eyes having smaller CA than RA. In addition, the preoperative magnitude difference between CA and RA with ME showed a significant relationship, implying that astigmatism in eyes having larger CA than RA has a higher potential to be overcorrected than that of eyes having smaller CA than RA. Since Kanellopoulos [6] did not perform a vector analysis of astigmatic change, and no information on the magnitude difference between preoperative CA and RA was provided, the reason for the difference in distribution of postoperative residual astigmatism between the two studies needs to be further investigated. Meanwhile, although the current treatment guideline of Contoura™ Vision recommends that the correction target of a cylinder be based on the midpoint between CA and RA in eyes having a larger CA than RA, no convincing evidence has been released to support this recommendation. Considering our results showing astigmatic overcorrection of eyes having a larger preoperative CA than RA in TMR-LASIK, the midpoint correction target could be speculated to also overcorrect astigmatism although the degree of overcorrection would be smaller than that of TMR-LASIK.

In our study, the increase in corneal HOAs after manifest TG- and TMR-LASIK was less than that after WFO-LASIK. We found that total HOA and coma were lower in both the TMR group and the TG group compared with the WFO group. Previous studies that have evaluated HOAs after LASIK have also shown that manifest TG-LASIK induced significantly fewer HOAs. Shetty et al. [10] reported that corneal spherical aberration was lower in manifest TG-LASIK than in WFO-LASIK in their contralateral-eye comparison. Other contralateral-eye comparative studies [3, 4] have also reported that manifest TG-LASIK induced less ocular coma, spherical aberration, and higher cylindrical aberrations.

The major strength of this study was that we first identified overcorrection of astigmatism after TMR ablation by conducting vector analysis of astigmatic change. Also, by subgroup analysis of the TMR group, together with a correlation analysis and multivariable linear regression, we suggested the magnitude difference between preoperative CA and RA as a putative reason for the astigmatic overcorrection after TMR-LASIK. Thus, our study provides eligibility criteria to select an appropriate ablation profile for LASIK surgery according to the preoperative astigmatism value. However, the present study also had limitations, including its retrospective nature, a short-term follow-up, and a relatively small sample size.

## Conclusions

In conclusion, although TMR-LASIK induces fewer corneal HOAs than conventional ablation, the possibility of astigmatic overcorrection after TMR-LASIK should be entertained. Future prospective studies that evaluate the effect of the magnitude difference between preoperative CA and RA on the surgical outcomes of TMR-LASIK, with a longer follow-up and a larger number of patients are warranted to develop a truly customized LASIK procedure.

## Data Availability

The datasets used and analyzed during the current study are available from the corresponding author on reasonable request.

## Abbreviations

LASIK: Laser in situ keratomileusis
TMR: Topography-modified refraction
WFO: Wavefront-optimized
TG: Topography-guided
UDVA: Uncorrected distance visual acuity
BCVA: Best-corrected distance visual acuity
MRSE: Manifest refraction spherical equivalent
D: Diopters
HOA: Higher-order aberrations
FDA: Food and Drug Administration
RA: Refractive astigmatism
CA: Corneal astigmatism
IRB: Institutional review board
TIA: Target-induced astigmatism
SIA: Surgically-induced astigmatism
DV: Difference vector
ME: Magnitude of error
CI: Correction index
IOS: Index of success
SD: Standard deviation
ANOVA: Analysis of variance
