# Correction to: Comparison of outcomes after topography-modified refraction versus wavefront-optimized versus manifest topography-guided LASIK

**DOI:** 10.1186/s12886-020-01753-x

**Published:** 2020-12-29

**Authors:** Jaeryung Kim, Sung-Ho Choi, Dong Hui Lim, Gil-Joong Yoon, Tae-Young Chung

**Affiliations:** 1 Department of Ophthalmology, Samsung Medical Center, Sungkyunkwan University School of Medicine, Seoul, Republic of Korea; 2 BALGEUN-EYE21 Operation Center, Gwangju, Republic of Korea

**Correction to: BMC Ophthalmol (2020) 20:192**

**https://doi.org/10.1186/s12886-020-01459-0**

Following publication of the original article [1], we were notified that the paper confirmed a previous study by Wallerstein et al. [2] and that this study was not cited in the published manuscript. The citation is included below.

Original:

- Our results showed that the predictability of the refractive correction and visual outcomes did not differ significantly among WFO-, manifest TG-, and TMR-LASIK, similar with those of previous contralateral-eye comparisons of WFO-LASIK with manifest TG- or TMR-LASIK [3–5]. However, in this study, the mean value of postoperative RA was higher in TMR-LASIK, and TMR-LASIK showed a significantly-skewed distribution of postoperative RA toward higher astigmatic values than WFO- and manifest TG-LASIK. On the other hand, a previous comparison between WFO- and TMR-LASIK reported conflicting results with respect to RA [5].

Corrected:

- Our results showed that the predictability of the refractive correction and visual outcomes did not differ significantly among WFO-, manifest TG-, and TMR-LASIK, similar with those of previous contralateral-eye comparisons of WFO-LASIK with manifest TG- or TMR-LASIK [3–5]. However, in this study, the mean value of postoperative RA was higher in TMR-LASIK, and TMR-LASIK showed a significantly-skewed distribution of postoperative RA toward higher astigmatic values than WFO- and manifest TG-LASIK. Similar results were previously reported by Wallerstein et al. [2] who found that in cases of eyes having a RA versus CA axis difference higher than 20°, eyes treated with TMR-LASIK had higher postoperative RA compared to those treated with manifest TG-LASIK. On the other hand, a previous comparison between manifest TG- and TMR-LASIK reported conflicting results with respect to postoperative RA [5].
